# Factors affecting on the mental development in children with Congenital Hypothyroidism

**DOI:** 10.1186/1687-9856-2013-S1-P147

**Published:** 2013-10-03

**Authors:** Nguyen Phu Dat, Van Lam Tra, Vu Chi Dung, Bui Phuong Thao, Can Thi Bich Ngoc, Nguyen Ngoc Khanh, Nguyen Thi Hoan

**Affiliations:** 1 Ha Noi Medical University, Hanoi, Vietnam; 2 National Hospital of Pediatrics, Hanoi, Vietnam

## 

Congenital hypothyroidism (CHT) is a common endocrine disease in children. The disease should be monitored and required longlife hormone replacement therapy. Is necessary to assess the metal status and affecting factors to help in management and long-term follow-up for children with CHT. So we review the factors which affect the mental development in children with CHT. The study recruited 70 children who were diagnosed CHT in National Hospital of Pediatrics from August 2001 to August 2011. It was a descriptive and cohort study. In this study, children were treated before 3 months of age have mental development as well as healthy children at the same age. In children diagnosed and treated between 3 months to <12 months of age, the risk of mental retardation was increased by 13.5 times. If treatment from 12 months to < 60 months of age, the risk of mental retardation was greater than 28. 8 times. If treatment after 60 months of age, the risk was 64 times higher than that of healthy children. Children without adherence to CHT medication had 4.1 times higher risk of mental retardation compared to children with adherence of CHT medication. These results suggest that the factors affecting CHT treatment outcomes were early diagnosis and treatment as well as treatment adherence.

